# Dynamics and Information Import in Recurrent Neural Networks

**DOI:** 10.3389/fncom.2022.876315

**Published:** 2022-04-27

**Authors:** Claus Metzner, Patrick Krauss

**Affiliations:** ^1^Neuroscience Lab, University Hospital Erlangen, Erlangen, Germany; ^2^Cognitive Computational Neuroscience Group, Friedrich-Alexander-University Erlangen-Nuremberg, Erlangen, Germany; ^3^Pattern Recognition Lab, Friedrich-Alexander-University Erlangen-Nuremberg, Erlangen, Germany

**Keywords:** recurrent neural networks (RNNs), dynamical system, edge of chaos, information processing, resonance phenomena

## Abstract

Recurrent neural networks (RNNs) are complex dynamical systems, capable of ongoing activity without any driving input. The long-term behavior of free-running RNNs, described by periodic, chaotic and fixed point attractors, is controlled by the statistics of the neural connection weights, such as the density *d* of non-zero connections, or the balance *b* between excitatory and inhibitory connections. However, for information processing purposes, RNNs need to receive external input signals, and it is not clear which of the dynamical regimes is optimal for this information import. We use both the average correlations *C* and the mutual information *I* between the momentary input vector and the next system state vector as quantitative measures of information import and analyze their dependence on the balance and density of the network. Remarkably, both resulting phase diagrams *C*(*b, d*) and *I*(*b, d*) are highly consistent, pointing to a link between the dynamical systems and the information-processing approach to complex systems. Information import is maximal not at the “edge of chaos,” which is optimally suited for computation, but surprisingly in the low-density chaotic regime and at the border between the chaotic and fixed point regime. Moreover, we find a completely new type of resonance phenomenon, which we call “Import Resonance” (IR), where the information import shows a maximum, i.e., a peak-like dependence on the coupling strength between the RNN and its external input. IR complements previously found Recurrence Resonance (RR), where correlation and mutual information of successive system states peak for a certain amplitude of noise added to the system. Both IR and RR can be exploited to optimize information processing in artificial neural networks and might also play a crucial role in biological neural systems.

## Introduction

At present, the field of Machine Learning is strongly dominated by feed-forward neural networks, which can be optimized to approximate an arbitrary vectorial function **y** = **f**(**x**) between the input and output spaces (Funahashi, 1989; Hornik et al., 1989; Cybenko, 1992). Recurrent neural networks (RNNs) however, are a much broader class of models, which encompass the feed-forward architectures as a special case, but which also include partly recurrent systems, such as contemporary LSTMs (long short-term memories) (Hochreiter and Schmidhuber, 1997) and classical Jordan or Elman networks (Cruse, 2006), up to fully connected systems without any layered structure, such as Hopfield networks (Ilopfield, 1982) or Boltzmann machines (Hinton and Sejnowski, 1983). Due to the feedback built into these systems, RNNs can learn robust representations (Farrell et al., 2019), and are ideally suited to process sequences of data such as natural language (LeCun et al., 2015; Schilling et al., 2021a), or to perform sequential-decision tasks such as spatial navigation (Banino et al., 2018; Gerum et al., 2020). Furthermore, RNNs can act as autonomous dynamical systems that continuously update their internal state **s**_*t*_ even without any external input (Gros, 2009), but it is equally possible to modulate this internal dynamics by feeding in external input signals **x**_*t*_ (Jaeger, 2014). Indeed, it has been shown that RNNs can approximate any open dynamical system **s**_*t*+1_ = **g**(**s**_*t*_, **x**_*t*_) to arbitrary precision (Schäfer and Zimmermann, 2006).

It is therefore not very surprising that biological neural networks are also highly recurrent in their connectivity (Binzegger et al., 2004; Squire et al., 2012), so that RNN models play an important role in neuroscience research as well Barak (2017) and Maheswaranathan et al. (2019). Modeling natural RNNs in a realistic way requires the use of probabilistic, spiking neurons, but even simpler models with deterministic neurons already have highly complex dynamical properties and offer fascinating insights into how structure controls function in non-linear systems (Krauss et al., 2019b,c). For example, we have demonstrated that by adjusting the density *d* of non-zero connections and the balance *b* between excitatory and inhibitory connections in the RNN's weight matrix, it is possible to control whether the system will predominantly end up in a periodic, chaotic, or fixed point attractor (Krauss et al., 2019b). Understanding and controlling the behavior of RNNs is of crucial importance for practical applications (Haviv et al., 2019), especially as meaningful computation, or information processing, is believed to be only possible at the “edge of chaos” (Bertschinger and Natschläger, 2004; Natschläger et al., 2005; Legenstein and Maass, 2007; Schrauwen et al., 2009; Büsing et al., 2010; Toyoizumi and Abbott, 2011; Dambre et al., 2012).

In this paper, we continue our investigation of RNNs with deterministic neurons and random, but statistically controlled weight matrices. Yet, the present work focuses on another crucial precondition for practical RNN applications: the ability of the system to store information, i.e., to “take up” external information and to incorporate it into the ongoing evolution of the internal system states. For this purpose, we first set up quantitative measures of information import, in particular the input-to-state correlation *C*(**x**_*t*_, **s**_*t*+1_), which is defined as the root-mean-square (RMS) average of all pairwise neural correlations between the momentary input **x**_*t*_ and the subsequent system state **s**_*t*+1_. Furthermore, we compute the input-to-state mutual information *I*(**x**_*t*_, **s**_*t*+1_), an approximation for the mean pairwise mutual information between the same two quantities. We then compute these measures for all possible combinations of the structural parameters *b* (balance) and *d* (density) on a grid, resulting in high-resolution phase diagrams *C*(*b, d*) and *I*(*b, d*). This reveals that the regions of phase space in which information storage (memory capacity) and information import (representation) are optimal, surprisingly do not coincide, but nevertheless have a small area of phase space in common. We speculate that this overlap region, where both crucial functions are simultaneously possible, may represent a “sweet spot” for practical RNN applications and might therefore be exploited by biological nervous systems.

## Results

### Free-Running Network

**Figure 1:**
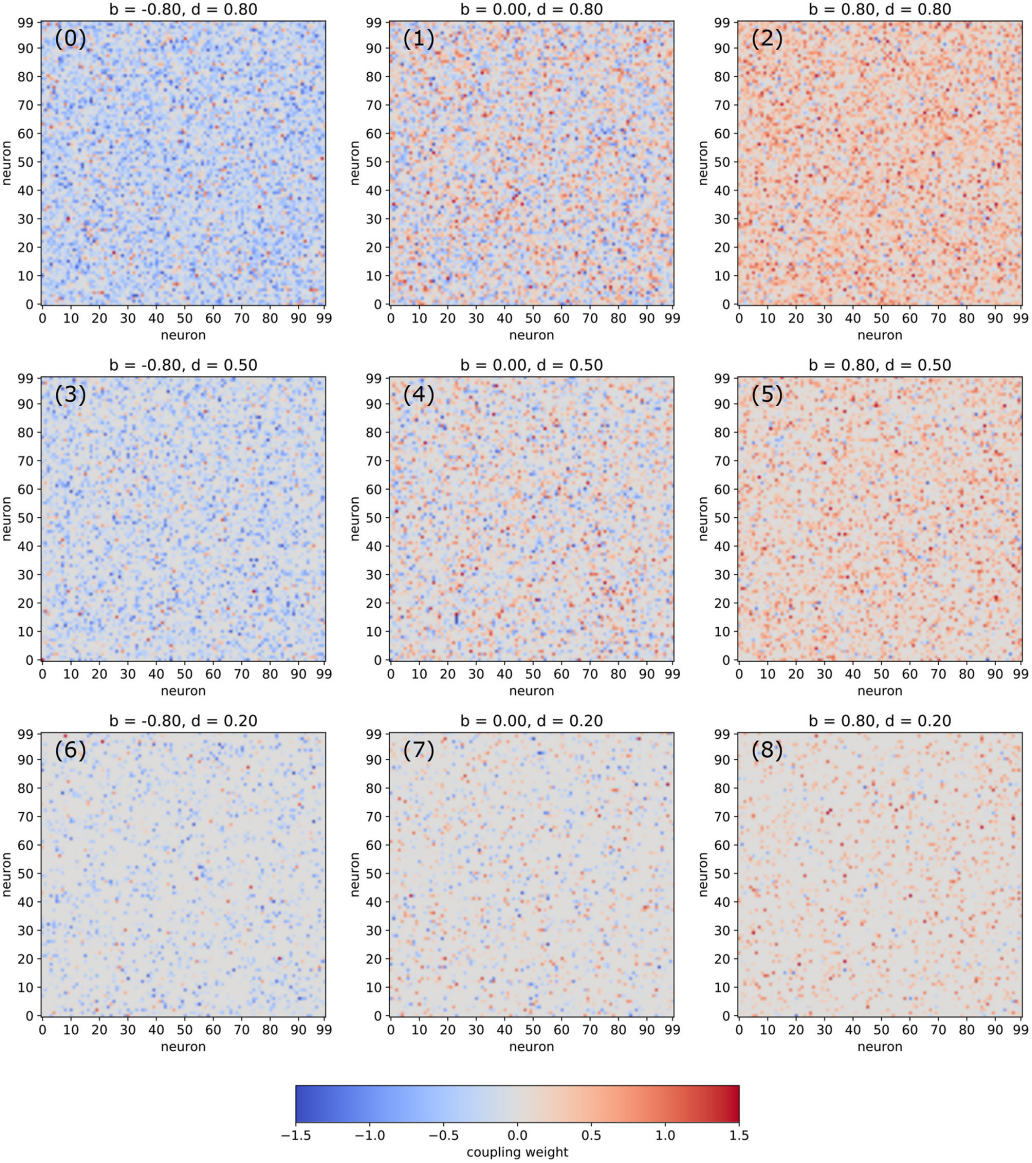
Examples of weight matrices for selected combinations of the balance *b* between excitatory and inhibitory connections and the density *d* of non-zero connections in an RNN.

In the following, we are analyzing networks composed of *N*_*neu*_ = 100 deterministic neurons with arctangent activation functions. The random matrix of connection weights is set up in a controlled way, so that the density *d* of non-zero connections as well the balance *b* between excitatory and inhibitory connections can be pre-defined independently (for details see Section 4). Visualizations of typical weight matrices for different combinations of the statistical control parameters *d* and *b* are shown in Figure 1.

**Figure 2:**
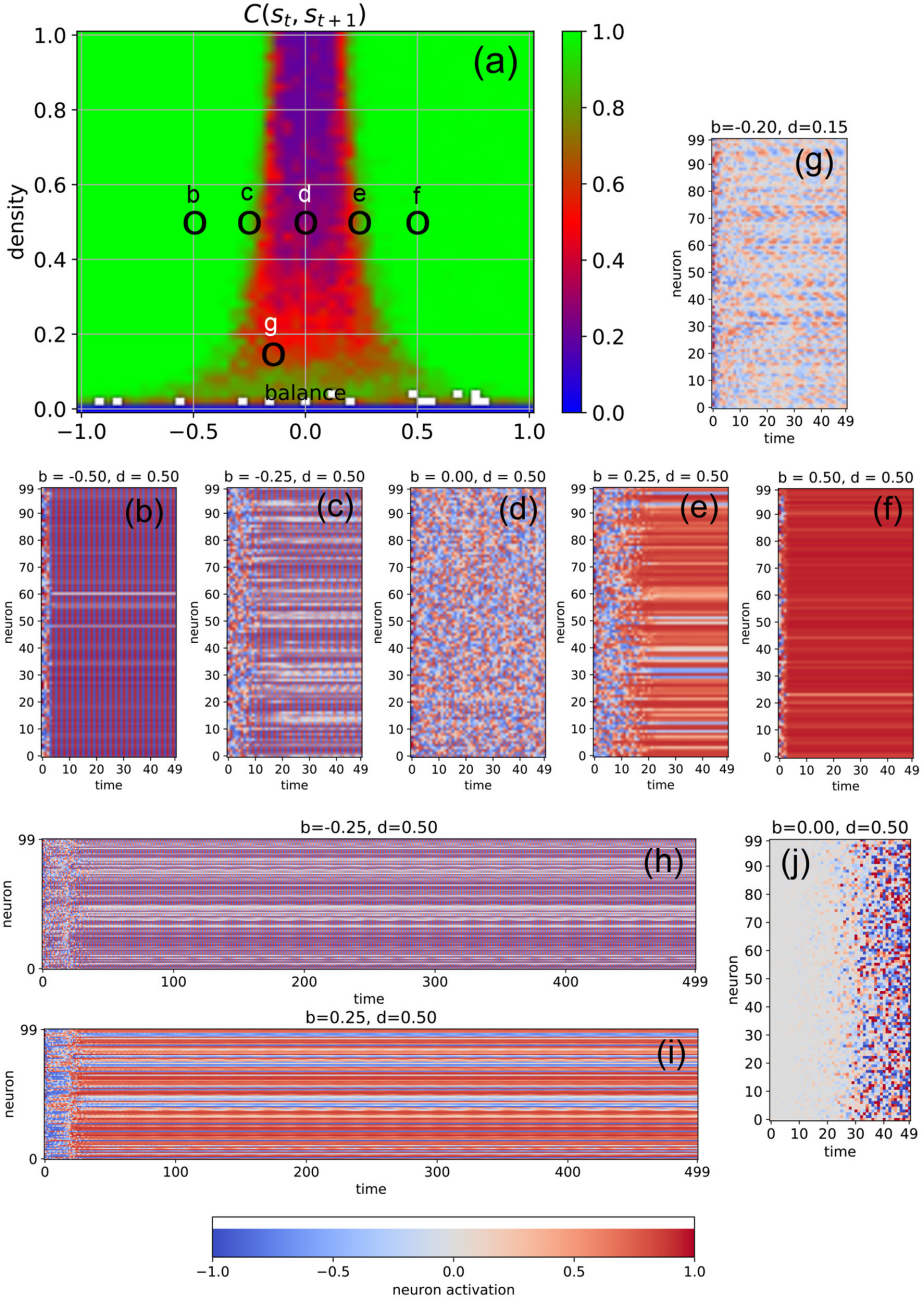
Dynamical phases of a free-running RNN, controlled by the structural parameters *b* (balance) and *d* (density). **(a)** Phase diagram of the correlation *C*(**s**_*t*_, **s**_*t*+1_) between successive neuron activations, as defined in the methods section. The three basic regimes are the oscillatory phase for negative balances (large correlations), the chaotic phase for balances close to zero (small correlations), and the fixed point phase for positive balances (large correlations). **(b–f)** Typical time dependence of the neural activations for fixed density (*d* = 0.5) and balances increasing from *b* = −0.5 to *b* = +0.5. The system behavior evolves from almost homogeneous oscillations **(b)**, to a heterogeneous oscillatory state **(c)**, to fully chaotic behavior **(d)**, to a heterogeneous fix point state state with a sub-group of slowly oscillating neurons **(e)**, and finally to an almost global fixed point attractor **(f)**. The low-density example **(g)** shows out-of-phase, imperfect oscillations with a period larger than 2, with phase differences between the neurons. Longer state sequences of the cases **(c,d)** are shown in **(h,i)**. **(j)** Shows the difference of neural activations between the chaotic state sequence **(d)** and a second run, where the initial activation of only one neuron (with index 0) was changed by a value of 0.1.

We first investigate free-running networks without external input and compute a dynamical phase diagram *C*_*ss*_(*b, d*) of the average correlation *C*_*ss*_ = *C*(**s**_*t*_, **s**_*t*+1_) between subsequent system states (Figure 2a, for details see Section 4). The resulting landscape is mirror-symmetric with respect to the line *b* = 0, due to the symmetric activation functions of our model neurons, combined with the definition of the balance parameter. Apart from the region of very low connection densities with *d* ≤ 0.1, the phase space consists of three major parts: the oscillatory regime in networks with predominantly inhibitory connections (*b* ≪ 0, left green area in Figure 2a), the chaotic regime with approximately balanced connections (*b* ≈ 0, central blue and red area in Figure 2a), and the fixed point regime with predominantly excitatory connections (*b* ≫ 0, right green area in Figure 2a).

It is important to note that *C*(**s**_*t*_, **s**_*t*+1_) is a root-mean-square (RMS) average over all the *N*_*neu*_ × *N*_*neu*_ pairwise correlations between subsequent neural activations (so that negative and positive correlations are not distinguished), and that these pairwise correlations are properly normalized in the sense of a Pearson coefficient (each ranging between –1 and +1 before the RMS is computed). For this reason, *C*(**s**_*t*_, **s**_*t*+1_) is close to one (green) both in the oscillatory and in the fixed point regimes, where the system is behaving regularly. By contrast, *C*(**s**_*t*_, **s**_*t*+1_) is close to zero (blue) in the high-density part of the chaotic regime, where the time-evolution of the system is extremely irregular. Medium-level correlations (red) are therefore expected in the transition region between these two extreme dynamical regimes, and they are indeed found in the correlation phase diagram for densities larger than ≈0.3 in the form of narrow stripes at the border of the chaotic “valley.” It is however surprising that medium-level correlations also exist across the whole chaotic valley for relatively low densities *d* ∈ [0.1, 0.3]. Since medium-level correlations are thought to be optimally suited for information processing (Bertschinger and Natschläger, 2004; Natschläger et al., 2005; Legenstein and Maass, 2007; Schrauwen et al., 2009; Büsing et al., 2010; Toyoizumi and Abbott, 2011; Dambre et al., 2012), it is remarkable that this can take place not only at the classical “edge of chaos” (between the oscillatory and the chaotic regime), but also in other (and less investigated) regions of the network's dynamical phase space.

In order to verify the nature of the three major dynamical regimes, we investigate the time evolution of the neural activations for selected combinations of the control parameters *b* and *d*. In particular, we fix the connection density to *d* = 0.5 and gradually increase the balance from *b* = −0.5 to *b* = +0.5 in five steps (Figures 2b–f). As expected, we find almost perfect oscillations (here with a period of two time steps) for *b* = −0.5 (case Figure 2b), at least after the transient period in which the system is still carrying a memory of the random initialization of the neural activations. At *b* = 0 (case Figure 2d), we find completely irregular, chaotic behavior, and at *b* = +0.5 (case Figure 2d) almost all neurons reach the same fixed point. However, the cases close to the two edges of the chaotic regime reveal an interesting intermediate dynamic behavior: For *b* = −0.25 (cases Figures 2c,h), most neurons are synchronized in their oscillations, but some are out of phase. Others show a long-period regular “beating”-like behavior superposed on the oscillations of period two (see the longer time trace in Figure 2h). For *b* = +0.25 (cases Figures 2e,i), most neurons reach (approximately) a shared fixed point, but some end up in a different, individual fixed point, thus resembling a state of quenched disorder. However, a sub-group of neurons is simultaneously engaged in long-period oscillations (see the longer time trace in Figure 2i).

The apparent irregularity of the neural activations in case Figure 2d does not necessarily imply chaotic behavior. To demonstrate the sensitive dependence of the neural trajectories on the initial condition, we change the activation of only a single neuron at *t* = 0 by a small amount of 0.1 and re-run the simulation. We find that drastic, system-spanning differences appear between the two time evolutions after about 30 time steps (see Figure 2j).

Moreover, we observe that the memory time τ of the system for the information imprinted by the initialization (that is, the duration of the transient phase) depends systematically on the balance parameter: Deep within the oscillatory regime (*b* = −0.5, case Figure 2b), τ is short. As we approach the chaotic regime (*b* = −0.25, case Figure 2c), τ increases, finally becoming “infinitely” long at *b* = 0 (case Figure 2d). Indeed, from this viewpoint the chaotic dynamics may be interpreted as the continuation of the transient phase. As we move deeper into the fixed point regime (cases Figures 2e,f), the memory time τ is decreasing again.

In the medium and high-density regime of the phase diagram, we find for negative values of the bias parameter mainly oscillations of period two, as the large number of negative weights causes the neurons to switch the sign of their sigmoidal outputs from one time step to the next. However, in the low-density regime, the magnitude of the neuron's total input is reduced and we then find also oscillations with larger periods (case Figure 2g).

### Network Driven by Continuous Random Input

Next, we feed into the network a relatively weak external input (with a coupling strength of η = 0.5), consisting of independent normally distributed random signals that are continuously injected to each of the neurons (for details see Section 4).

**Figure 3:**
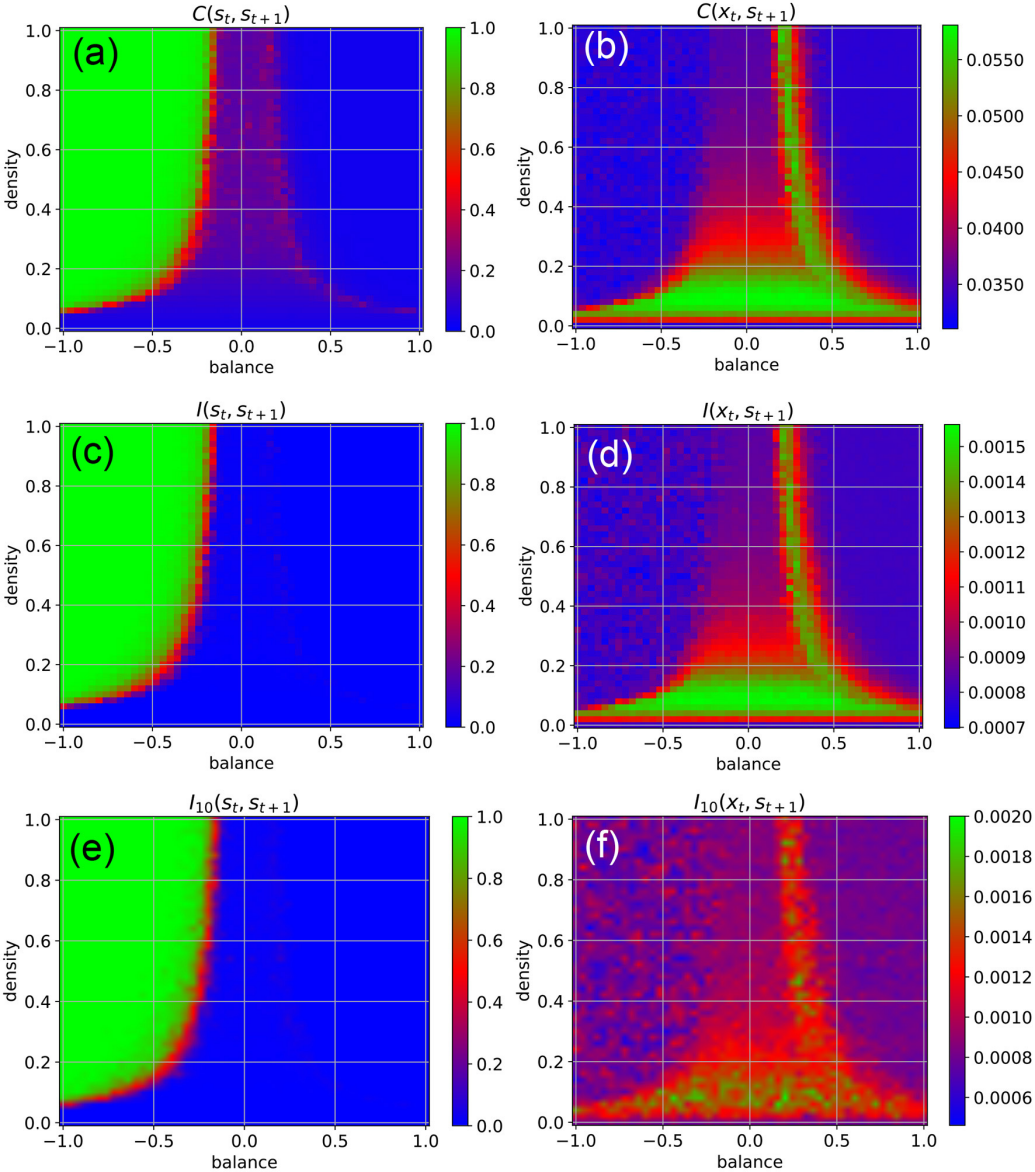
Dynamical phases of a RNN driven by external input in the form of continuous random signals that are coupled independently to all neurons with a coupling constant of η = 0.5. The suitability of the system for information processing is characterized by the statistical dependency between subsequent states (left column), the suitability for information import by the statistical dependency between the input **x**_*t*_ and the subsequent state **s**_*t*+1_ (right column). First row **(a,b)** Root-mean-square of correlations. Second row **(c,d)** Mean pairwise mutual information. Information import is optimal in the low-density chaotic regime and at the border between the chaotic and fixed point regime (red and green color in right column). Third row **(e,f)** Approximation of the mean pairwise mutual information, where only a sub-population of 10 neurons is included to the evaluation.

We find that the external input destroys the medium-level state-to-state correlations *C*(**s**_*t*_, **s**_*t*+1_) in most parts of the chaotic regime, except at the classical edge of chaos (Figure 3a, red). Moreover, the input also brings the state-to-state correlations in the fixed point regime down to a very small value, as now the external random signals are superimposed onto the fixed points of the neurons.

Another important practical factor is the ability of neural networks to store information, i.e., to take up external information at any point in time and to incorporate it into their system state. We quantify this ability of information import by the RMS-averaged correlation *C*(**x**_*t*_, **s**_*t*+1_) between momentary input and subsequent system state. Surprisingly, we find that information import is best, i.e., *C*(**x**_*t*_, **s**_*t*+1_) is large, in the low-density part of the chaotic regime, including the lowest part of the classical edge of chaos (region between chaotic and oscillatory regimes), but also at the opposite border between the chaotic and fixed point regimes (Figure 3b, green and red). We thus come to the conclusion that (at least for weak external inputs with η = 0.5) our network model is simultaneously capable of information import and information processing only in the low-density part of the classical edge of chaos.

To backup this unexpected finding, we also quantify information storage and information import by the average pair-wise state-to-state mutual information *I*(**s**_*t*_, **s**_*t*+1_) (Figure 3c), and the mutual information between the momentary input and the subsequent system state *I*(**x**_*t*_, **s**_*t*+1_) (Figure 3d), respectively. These mutual-information-based measures can also capture possible non-linear dependencies, but are computationally much more demanding (for details see Section 4).

Despite of these drastic differences between the two measures, we obtain practically the same phase diagrams for information import and information storage/processing when we use the RMS-averaged pairwise correlations (Figures 3a,b) and when we use the mutual information (Figures 3c,d). This congruence may simply indicate the absence of higher-order statistical dependencies between subsequent states in our specific RNN system. However, in the context of adaptive stochastic resonance, we have already reported a surprisingly close relation between linear correlation and mutual information for a large range of model systems (Krauss et al., 2017). Taken together, these findings suggest a possible link between information-processing and dynamical approaches to complexity science (Mediano et al., 2021).

Furthermore, we compare the results to a computationally more tractable approximation of the mean pairwise mutual information, where only a sub-population of 10 neurons is included to the evaluation. It also shows the same basic characteristics (Figures 3e,f), implicating the possibility to approximate mutual information in large dynamical systems, where an exhaustive sampling of all joint probabilities necessary to calculate entropy and mutual information is impractical or impossible.

### Effect of Other System Parameters

**Figure 4:**
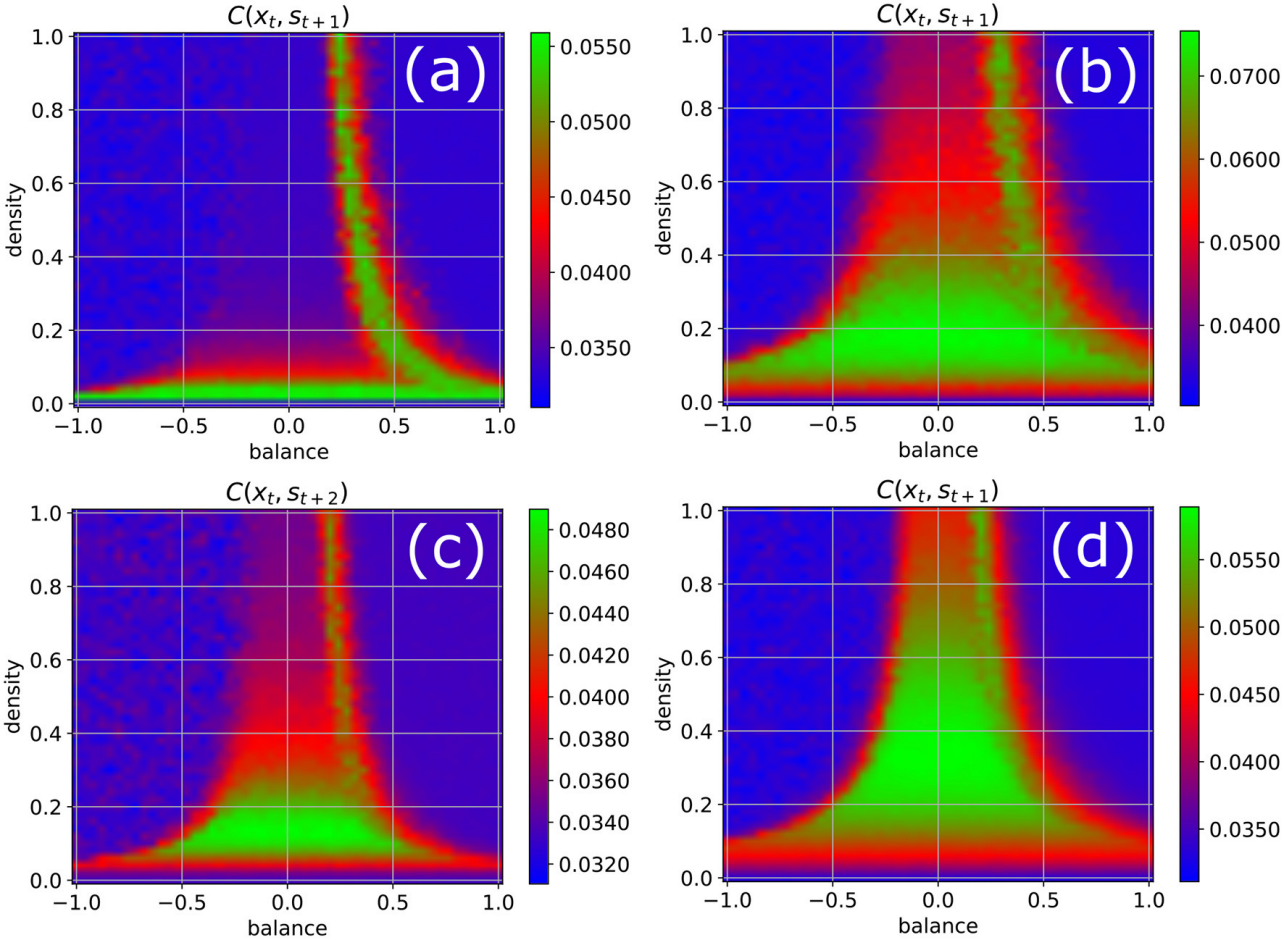
Phase diagram of information import as in Figure 3b, but with one parameter changed in each of the four panels. **(a)** Width of the Gaussian distribution of weight magnitudes increased from *w* = 0.5 to *w* = 1. **(b)** Number of neurons reduced from *N* = 100 to *N* = 50. **(c)** Time delay between input signal and system state increased from 1 to 2. **(d)** Width of the Gaussian distribution of weight magnitudes decreased from *w* = 0.5 to *w* = 0.25. The results are similar to Figure 3b in all cases except for reduced weight fluctuations **(d)**, where both edges of chaos become available for information import.

In order to test the robustness of the above results on information import, we re-compute the phase diagram of the correlations between the input and a later system state (Figure 4), now however varying some of the parameters that have been kept at their standard values (*w* = 0.5, *N* = 100, Δ*t* = 1, η = 0.5) so far. We obtain results similar to Figure 3b when the fluctuation width *w* of the Gaussian weight distribution is increased to *w* = 1 (Figure 4a), when the number of neurons in reduced to *N* = 50 (Figure 4b), and when the lag-time between input signal and system state is increased to Δ*t* = 2 (Figure 4c). However, when the fluctuation width of the weight distribution is reduced to *w* = 0.5, which decreases the total neural inputs and therefore brings the system closer to the linear regime, we find that now both edges of chaos become available for information uptake (Figure 4d).

### Effect of Increasing Coupling Strength

**Figure 5:**
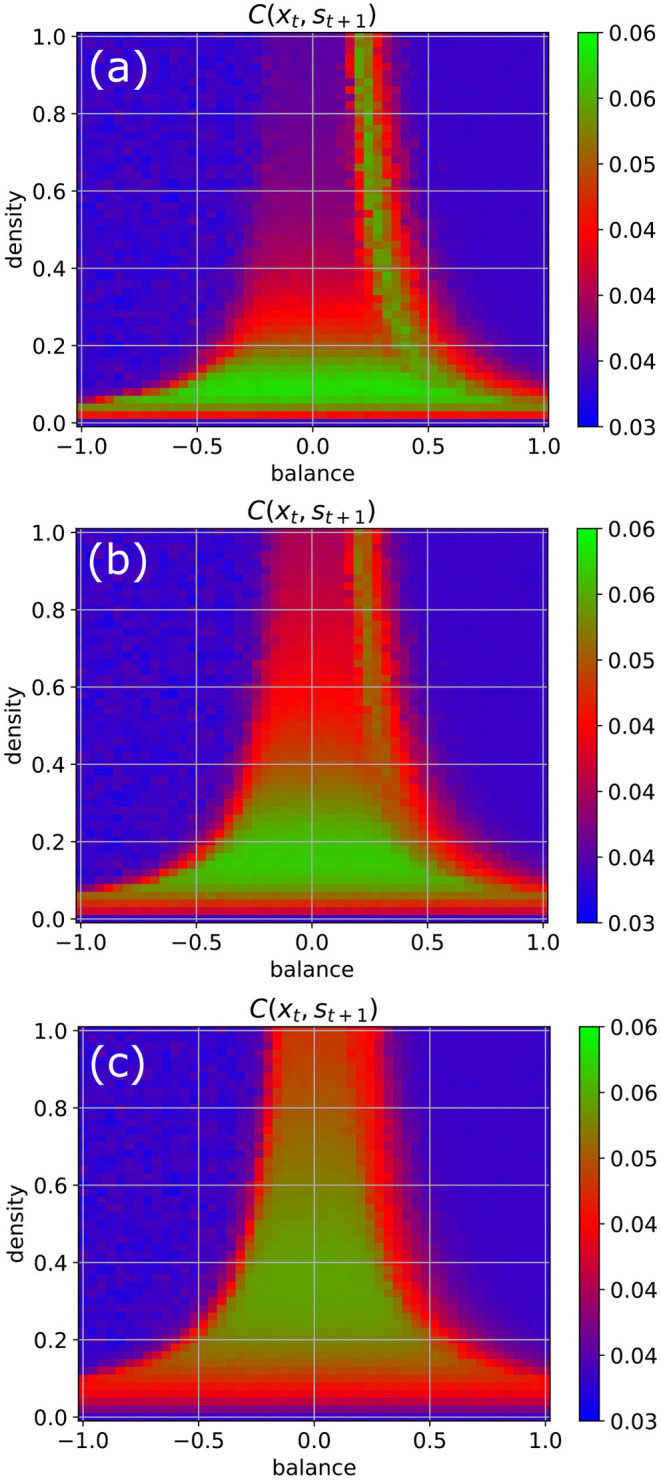
Information import as a function of the coupling strength η between the RNN neurons and the external input signals. For weak coupling [η = 0.5 in **(a)**], only the low-density chaotic regime and the border between the chaotic and fixed point regime are suitable for information import. As the coupling in increases from η = 1 in **(b)** to η = 2 in **(c)**, the correlations between input **x**_*t*_ and subsequent RNN states **s**_*t*+1_ become gradually large throughout the complete chaotic regime.

We return to our standard parameters (*w* = 0.5, *N* = 100, Δ*t* = 1), but now increase the coupling strength to the random input signals step-wise from η = 0.5 to η = 1 and finally to η = 2 (Figure 5). We observe that by this way also the higher density parts of the chaotic regime become eventually available for information import (green color).

### Import Resonance (IR) and Recurrence Resonance (RR)

Next, we increase the coupling strength η gradually from zero to a very large value of 20, at which the random input already dominates the system dynamics. For this numerical experiment, we keep the balance and density parameters fixed at *b* = −0.5, *d* = 0.5 (oscillatory regime), *b* = 0, *d* = 0.5 (chaotic regime), and *b* = 0.5, *d* = 0.5 (fixed point regime), respectively.

**Figure 6:**
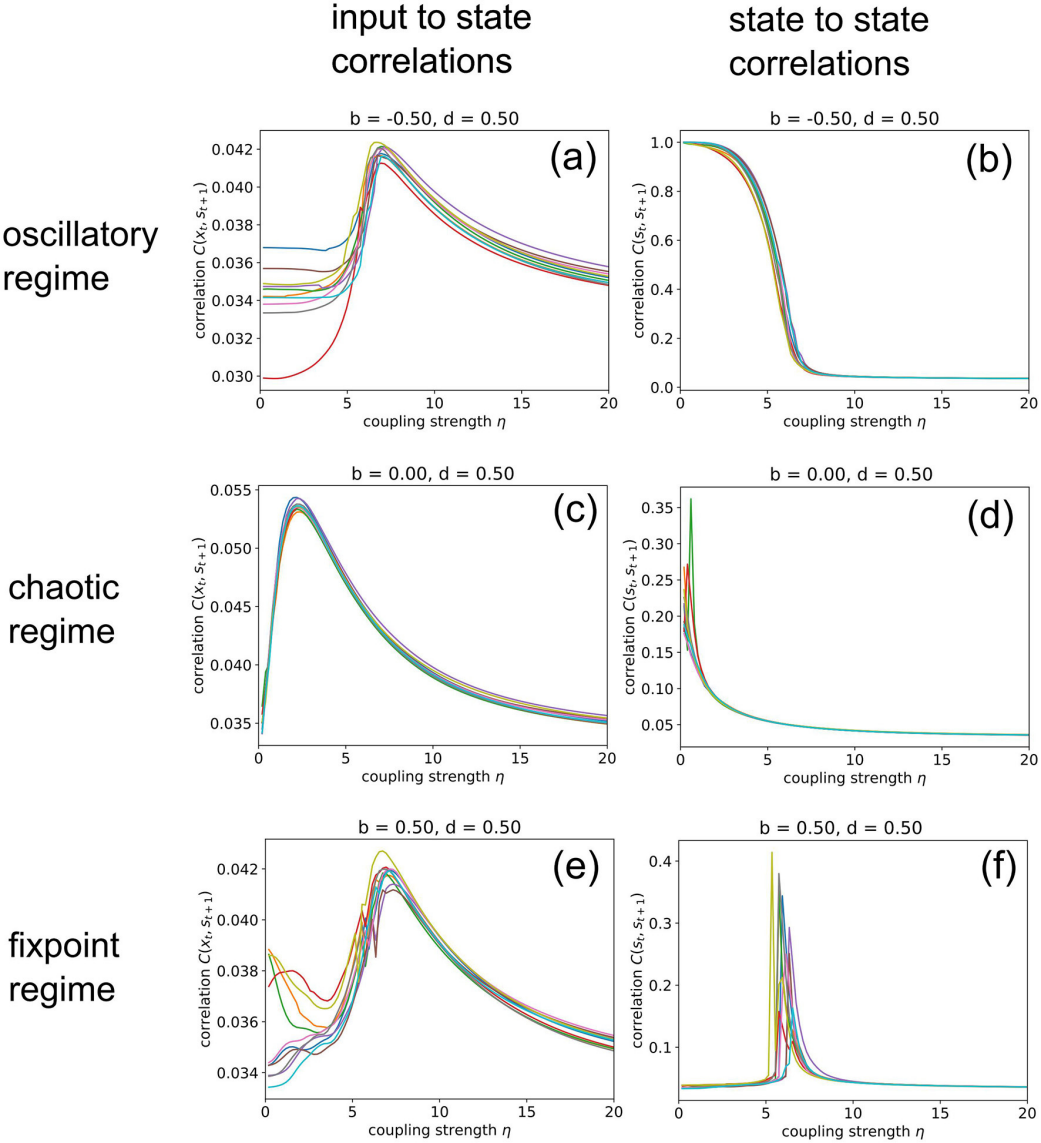
Import resonance and recurrence resonance in RNNs. We compute the input-to-state correlation *C*(**x**_*t*_, **s**_*t*+1_) (left column) and the state-to-state correlation *C*(**s**_*t*_, **s**_*t*+1_) (right column) for RNNs in the oscillatory (top row), chaotic (middle row) and fixed point regimes (bottom row), as the coupling strength to the random (noise) input **x**_*t*_ is gradually increased from zero to 20. The computation has been repeated for 10 different realizations (colors) of RNNs with the given control parameters *b* (balance) and *d* (density). We find the phenomenon of import resonance in all three dynamical regimes **(a,c,e)** and the phenomenon of recurrence resonance in the fixed point regime **(f)**. No resonance is found in cases **(b,d)**.

When in the fixed point regime (Figure 6f), we find that the dependence of the state-to-state correlation *C*(**s**_*t*_, **s**_*t*+1_) on the coupling strength η has the shape of a “resonance peak.” Since η effectively controls the amplitude of “noise” (used by us as pseudo input) added to the system, this corresponds to the phenomenon of “Recurrence Resonance” (RR), which we have previously found in three-neuron motifs (Krauss et al., 2019a): At small noise levels η, the system is stuck in the fixed point attractor, but adding an optimal amount of noise (so that *C*(**s**_*t*_, **s**_*t*+1_) becomes maximal) is freeing the system from this attractor and thus makes recurrent information “flux” possible, even in the fixed point regime. Adding too much noise is however counter-productive and leads to a decrease of *C*(**s**_*t*_, **s**_*t*+1_), as the system dynamics then becomes dominated by noise. We do not observe recurrence resonance in the other two dynamic regimes (Figures 6b,d).

Interestingly, we find very pronounced resonance-like curves also in the dependence of the input-to-state correlation *C*(**x**_*t*_, **s**_*t*+1_) on the coupling strength η, for all dynamical regimes (Figures 6a,c,e). Since *C*(**x**_*t*_, **s**_*t*+1_) is a measure of information import, we call this novel phenomenon “Import Resonance” (IR).

### Network Driven by Continuous Sinusoidal Input

**Figure 7:**
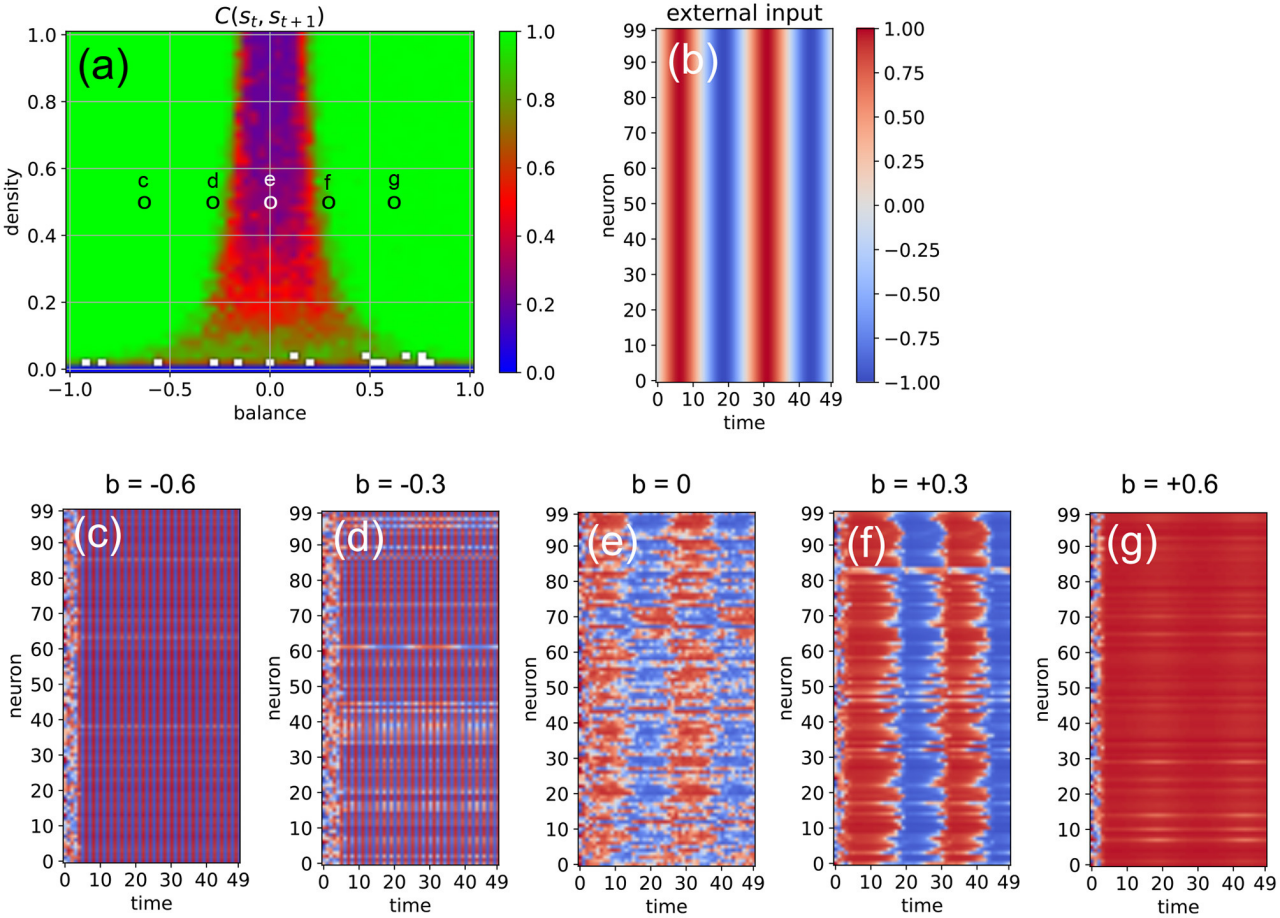
Effect of a “sinusoidal” input **(b)** on the activations of the RNN neurons **(c–g)** at five different points in the system's dynamic phase space **(a)**. For all cases **(c–g)**, the density parameter is *d* = 0.5, while the balance increases from –0.6 to +0.6. Only for balances sufficiently close to zero **(d,e,f)** the input is able to affect the system state.

Next, we investigate the ability of the system to import more regular input signals with built-in temporal correlations, as well as inputs that are identical for all neurons. For this purpose, we feed all neurons with the same sinusoidal input signal, using an amplitude of *a*_*sin*_ = 1, an oscillation period of *T*_*sin*_ = 25 time steps, and a coupling strength of η = 2 (Figure 7). The density parameter is again fixed at *d* = 0.5, while the balance increases from *b* = −0.6 to *b* = +0.6 in five steps. We find that the input signal does not affect the evolution of neural states when the system is too far in the oscillatory phase or too far in the fixed point phase (c,g). Only systems where excitatory and inhibitory connections are approximately balanced are capable of information import (d-f). For *b* = −0.3 (d), most of the neurons are still part of the periodic attractor, but a small sub-population of neurons is taking up the external input signal (d). Interestingly, the system state is reflecting the periodic input signal even in the middle of the chaotic phase (e).

### Correlations for Longer Lagtimes

**Figure 8:**
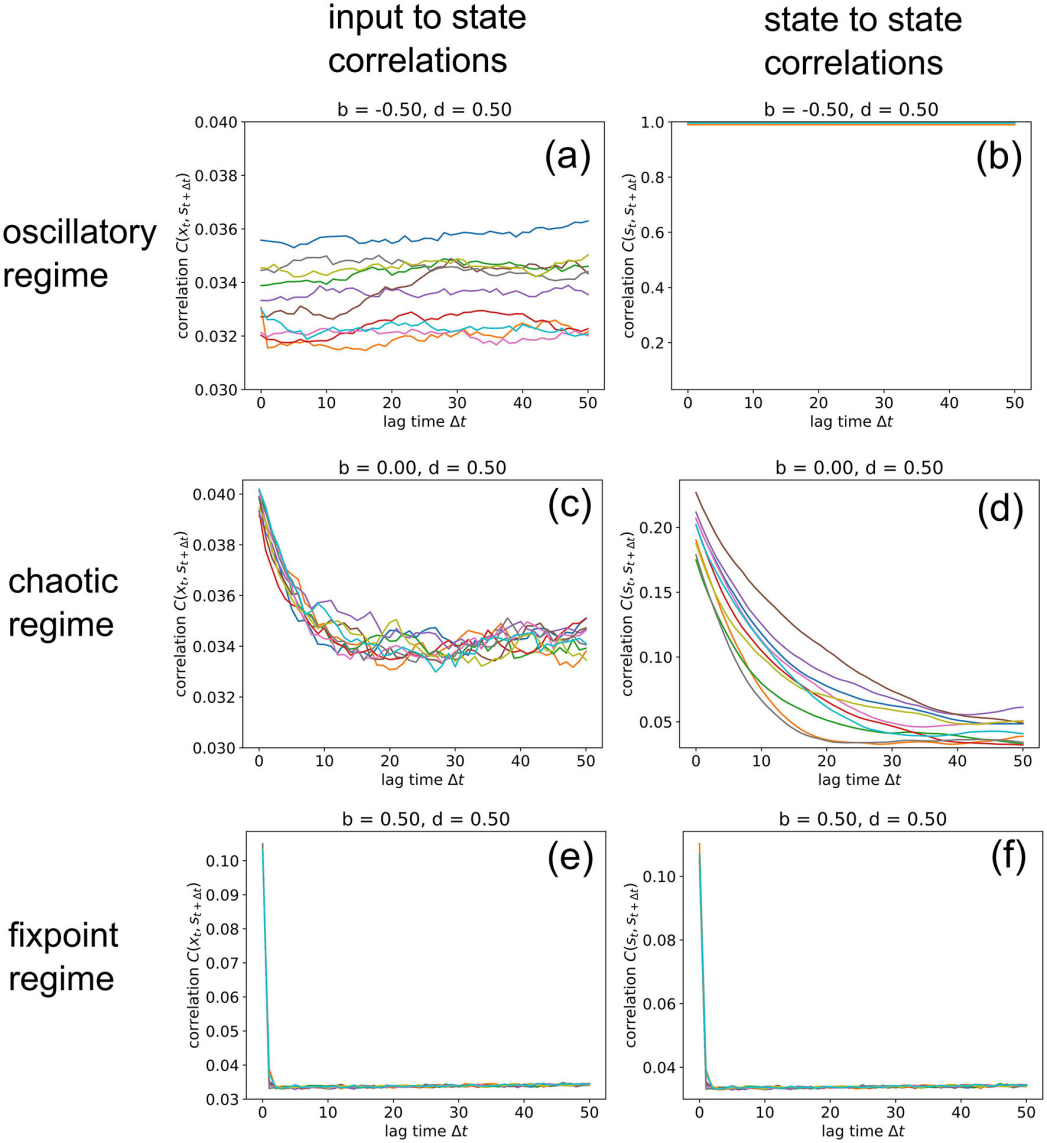
Information import and storage for longer lagtimes. We compute the input-to-state correlation *C*(**x**_*t*_, **s**_*t*+1_) (left column) and the state-to-state correlation *C*(**s**_*t*_, **s**_*t*+1_) (right column) for RNNs in the oscillatory (top row), chaotic (middle row) and fixed point regimes (bottom row), for increasing lagtimes between zero and 50. The computation has been repeated for 10 different realizations (colors) of RNNs with the given control parameters *b* (balance) and *d* (density). Note that correlations *C* never become lower than a noise level of about 0.034, because we compute *C* as an RMS average. Due to this RMS, the signature of an oscillatory state is *C*(**s**_*t*_, **s**_*t*+1_) = 1, as found in **(b)**. Import and storage of information, above the noise level (and at non-zero lagtimes), is observed only in the cases **(c,d)**, even though the RNN is deeply in the chaotic regime at *b* = 0, *d* = 0.5. In the oscillatory and fixpoint regimes **(a,b,e,f)**, this is not possible.

So far, we have analyzed input-to-state and state-to-state correlations mainly for a lag-time Δ*t* = 1. We finally extend this analysis to larger lag-times up to 50 time steps (Figure 8), however only for three selected RNNs in the oscillatory, chaotic and fixpoint regime, using again our standard parameters (*w* = 0.5, *N* = 100, η = 0.5). Since our correlation measures *C*(**x**_*t*_, **s**_*t*+1_) (left column) and *C*(**s**_*t*_, **s**_*t*+1_) (right column) are defined as RMS averages, these values never fall below a certain noise level, which is in our case about 0.034. Another consequence of the RMS-average is that perfectly oscillatory RNN states with a period of two show up as *C*(**s**_*t*_, **s**_*t*+1_) = 1 (Figure 8b).

In the oscillatory regime, we find that input-to state correlations (as a measure of information import) remain at the noise level for all lag-times (a), while the system states are bound in a perfectly periodic attractor (b). Also in the fixpoint regime, both types of correlation are negligible for all non-zero lag-times. But remarkably, information can be imported (c) and stored (d) to a small but significant extent even in the middle of the chaotic regime, although the correlations decay back to noise level after about 20 time steps for this specific point in phase space (*b* = 0, *d* = 0.5). Future work will analyze how this correlation decay time depends on the statistical system parameters *b* and *d*.

## Discussion

In this study, we investigate the ability of RNNs to import and store information as a function of the weight statistics, a problem that has been met with considerable interest during the past years (Bässler, 1986; Derrida et al., 1987; Gutfreund et al., 1988; Langton, 1990; Wang et al., 1990, 2011; Molgedey et al., 1992; Crisanti et al., 1993; Kaneko and Suzuki, 1994; Solé and Miramontes, 1995; Greenfield and Lecar, 2001; Jaeger, 2001; Bertschinger and Natschläger, 2004; Rajan et al., 2010; Toyoizumi and Abbott, 2011; Boedecker et al., 2012; Wallace et al., 2013; Kadmon and Sompolinsky, 2015; Brunel, 2016; Folli et al., 2018; Schuecker et al., 2018; Grigoryeva and Ortega, 2019; Grigoryeva et al., 2021). We specialize on discrete-time, deterministic RNNs with an arctan activation function and describe the weight statistics by the *density of non-zero weights* and on the *balance of excitatory and inhibitory connections*, as introduced in our previous studies (Krauss et al., 2019b,c). In contrast to the human brain, where the vast majority of neurons is either purely excitatory or purely inhibitory (Dale's principle), each given neuron can simultaneously have positive and negative output weights in our simplified model system.

It turned out that our RNN model is simultaneously capable of both information import and information storage only in the low-density, i.e., sparse, part of the classical edge of chaos. Remarkably, this region of the phase space corresponds to the connectivity statistics known from the brain, in particular the cerebral cortex (Song et al., 2005; Sporns, 2011; Miner and Triesch, 2016). In line with previous findings, i.e., that sparsity prevents RNNs from overfitting (Narang et al., 2017; Gerum et al., 2020) and is optimal for information storage (Brunel, 2016), we therefore hypothesize that cortical connectivity is optimized for both information import and processing. In addition, it seems plausible that there might be distinct networks in the brain that are either specialized to import and to represent information, or to process information and perform computations.

Furthermore, we found a completely new resonance phenomenon which we call *import resonance*, showing that the correlation or mutual information between input and the subsequent network state depends on certain control parameters (such as coupling strength) in a peak-like way. Resonance phenomena are ubiquitous not only in simplified neural network models (Ikemoto et al., 2018; Krauss et al., 2019a; Bönsel et al., 2021), but also in biologically more realistic systems (McDonnell and Abbott, 2009), where they show up in diverse variants such as coherence resonance (Lindner and Schimansky-Geier, 2000; Gu et al., 2002; Lindner et al., 2002), finite size resonance (Toral et al., 2003), bimodal resonance (Mejias and Torres, 2011; Torres et al., 2011), heterogeneity-induced resonance (Mejias and Longtin, 2012, 2014), or inverted stochastic resonance (Buchin et al., 2016; Uzuntarla et al., 2017). They have been shown to play a crucial role for neural information processing (Moss et al., 2004; Krauss et al., 2018; Schilling et al., 2020). In particular with respect to the auditory system, it has been argued that resonance phenomena like stochastic resonance are actively exploited by the brain to maintain optimal information processing (Krauss et al., 2016, 2017, 2018; Schilling et al., 2021b). For instance, in a theoretical study it could be demonstrated that stochastic resonance improves speech recognition in an artificial neural network as a model of the auditory pathway (Schilling et al., 2020). Very recently, we were even able to show that stochastic resonance, induced by simulated transient hearing loss, improves auditory sensitivity beyond the absolute threshold of hearing (Krauss and Tziridis, 2021). The extraordinary importance of resonance phenomena for neural information processing indicates that the brain, or at least certain parts of the brain, do also actively exploit other kinds of resonance phenomena besides classical stochastic resonance. Whereas, stochastic resonance is suited to enhance the detection of weak signals from the environment in sensory brain systems (Krauss et al., 2017), we speculate that parts of the brain dealing with sensory integration and perception might exploit import resonance, while structures dedicated to transient information storage (short-term memory) (Ichikawa and Kaneko, 2020) and processing might benefit from recurrence resonance (Krauss et al., 2019a). Similarly, the brain's action and motor control systems might also benefit from a hypothetical phenomenon of *export resonance*, i.e., the maximization of correlation or mutual information between a given network state and a certain, subsequent readout layer.

Finally, our finding that both, correlation- and entropy-based measures of information import and storage yield almost identical phase diagrams (Figures 3a,b compare with Figures 3c,d), is in line with previously published results, i.e., that mutual information between sensor input and output can be replaced by the auto-correlation of the sensor output in the context of stochastic resonance (SR) (Krauss et al., 2017). However, in this study we find that the equivalence of measures based on correlations and mutual information even extends to the phenomena of recurrence resonance (RR) (Krauss et al., 2019a) and import resonance (IR), thereby bridging the conceptual gap (as described in Mediano et al., 2021) between the information-processing perspective and the dynamical systems perspective on complex systems.

## Methods

### Weight Matrices With Pre-defined Statistics

We consider a system of *N*_*neu*_ neurons without biases, which are mutually connected according to a weight matrix {*w*_*mk*_}, where *w*_*mk*_ denotes the connection strength from neuron *k* to neuron *m*. The weight matrix is random, but controlled by three statistical parameters, namely the “*density*” *d* of non-zero connections, the excitatory/inhibitory “*balance*” *b*, and the “*width*” *w* of the Gaussian distribution of weight magnitudes. The density ranges from *d* = 0 (isolated neurons) to *d* = 1 (fully connected network), and the balance from *b* = −1 (purely inhibitory connections) to *b* = +1 (purely excitatory connections). The value of *b* = 0 corresponds to a perfectly balanced system.

In order to construct a weight matrix with given parameters (*b, d, w*), we first generate a matrix $M_{mn}^{\left(magn\right)}$ of weight magnitudes, by drawing the $N_{neu}^{2}$ matrix elements independently from a zero-mean normal distribution with standard deviation *w* and then taking the absolute value. Next, we generate a random binary matrix $B_{mn}^{\left(nonz\right)}\in \left\{0,1\right\}$, where the probability of a matrix element being 1 is given by the density *d*, i.e., *p*_1_ = *d*. Next, we generate another random binary matrix $B_{mn}^{\left(sign\right)}\in \left\{-1,+1\right\}$, where the probability of a matrix element being +1 is given by *p*_+1_ = (1 + *b*)/2 where *b* is the balance. Finally, the weight matrix is constructed by elementwise multiplication $w_{mn}=M_{mn}^{\left(magn\right)}\cdot \;B_{mn}^{\left(nonz\right)}\cdot \;B_{mn}^{\left(sign\right)}$. Note that throughout this paper, the width parameter is set to *w* = 0.5.

### Time Evolution of System State

The momentary state of the RNN is given by the vector **s**(*t*) = {*s*_*m*_(*t*)}, where the component *s*_*m*_(*t*) ∈ [−1, +1] is the activation of neuron *m* at time *t*. The initial state **s**(*t* = 0) is set by assigning to the neurons statistically independent, normally distributed random numbers with zero mean and a standard deviation of σ_*ini*_ = 1.

We then compute the next state vector by simultaneously updating each neuron *m* according to

(1) $$\begin{array}{r} s_{m}\left(t+1\right)=\frac{2}{\pi }\arctan\left(\eta \;x_{m}\left(t\right)+\overset{N}{\underset{k=1}{\sum }}w_{mk}s_{k}\left(t\right)\right). \end{array}$$

Here, *x*_*m*_(*t*) are the external inputs of the RNN and η is a global “*coupling strength”*. Note that the input time series *x*_*m*_(*t*) can, but must not be different for each neuron. In one type of experiment, we set the *x*_*m*_(*t*) to independent, normally distributed random signals with zero mean and unit variance. In another experiment, we set all *x*_*m*_(*t*) to the same oscillatory signal *x*(*t*) = *a*_*sin*_ · sin(2π*t*/*T*_*sin*_).

After simulating the sequence of system states for *N*_*stp*_ = 1000 time steps, we analyze the properties of the state sequence (see below). For this evaluation, we disregard the first *N*_*tra*_ = 100 time steps, in which the system may still be in a transitory state that depends strongly on the initial condition. The simulations are repeated *N*_*run*_ = 10 times for each set of control parameters (*b, d*, η).

### Root-Mean-Squared Pairwise Correlation *C*(**u**_*t*_, **v**_*t*+1_)

Consider a vector **u**(*t*) in *M* dimensions and a vector **v**(*t*) in *N* dimensions, both defined at discrete time steps *t*. The components of the vectors are denoted as *u*_*m*_(*t*) and *v*_*n*_(*t*). In order to characterize the correlations between the two time-dependent vectors by a single scalar quantity *C*(**u**_*t*_, **v**_*t*+1_), we proceed as follows:

First, we compute for each vector component *m* the temporal mean,

(2) $$\begin{array}{r} \mu_{um}={\langle u_{m}\left(t\right)\rangle }_{t} \end{array}$$

and the corresponding standard deviation

(3) $$\begin{array}{r} \sigma_{um}=\sqrt{{\langle {\left(u_{m}\left(t\right)-\mu_{um}\right)}^{2}\rangle }_{t}}. \end{array}$$

Based on this, we compute the *M* × *N* pairwise (Pearson) correlation matrix,

(4) $$\begin{array}{r} C_{mn}^{\left(uv\right)}=\frac{{\langle \left[u_{m}\left(t\right)-\mu_{um}\right]\cdot \left[v_{n}\left(t+1\right)-\mu_{vn}\right]\rangle }_{t}}{\sigma_{um}\sigma_{vn}}, \end{array}$$

defining $C_{mn}^{\left(uv\right)}=0$ whenever σ_*um*_ = 0 or σ_*vn*_ = 0.

Finally we compute the root-mean-squared average of this matrix,

(5) $$\begin{array}{r} C\left({\text{u}}_{t},{\text{v}}_{t+1}\right)=\text{RMS}{\left\{C_{mn}^{\left(uv\right)}\right\}}_{mn}=\sqrt{\frac{1}{MN}\overset{M}{\underset{m=1}{\sum }}\overset{N}{\underset{n=1}{\sum }}|C_{mn}^{\left(uv\right)}{|}^{2}} \end{array}$$

This measure is applied in the present paper to quantify the correlations *C*(**s**_*t*_, **s**_*t*+1_) between subsequent RNN states, as well as the correlations *C*(**x**_*t*_, **s**_*t*+1_) between the momentary input and the subsequent RNN state.

### Mean Pairwise Mutual Information *I*(**u**_*t*_, **v**_*t*+1_)

In addition to the linear correlations, we consider the mutual information between the two vectors **u**(*t*) and **v**(*t*), in order to capture also possible non-linear dependencies. However, since the full computation of this quantity is computationally extremely demanding, we binarize the continuous vector components and then consider only the pairwise mutual information between these binarized components.

For the binarization, we first subtract the mean values from each of the components,

(6) $$\begin{array}{r} u_{m}\left(t\right)\to \Delta u_{m}\left(t\right)=u_{m}\left(t\right)-\mu_{um}. \end{array}$$

We then map the continuous signals Δ*u*_*m*_(*t*) ∈ [−∞, +∞] onto two-valued bits $\hat{u}$_*m*_(*t*) ∈ {0, 1} by defining $\hat{u}$_*m*_(*t*) = 0 if Δ*u*_*m*_(*t*) < 0 and $\hat{u}$_*m*_(*t*) = 1 if Δ*u*_*m*_(*t*) > 0. In the case of a tie, Δ*u*_*m*_(*t*) = 0, we set $\hat{u}$_*m*_(*t*) = 0 with a probability of 1/2.

We next compute the pairwise joint probabilities $P\left({\hat{u}}_{m},{\hat{v}}_{n}\right)$ by counting how often each of the four possible bit combinations occurs during all available time steps. From that we also obtain the marginal probabilities *P*($\hat{u}$_*m*_) and $P\left({\hat{v}}_{n}\right)$.

The matrix of pairwise mutual information is then defined as

(7) $$\begin{array}{r} I_{mn}^{\left(uv\right)}=\underset{{\hat{u}}_{m}=0,1}{\sum }\underset{{\hat{v}}_{n}=0,1}{\sum }P\left({\hat{u}}_{m},{\hat{v}}_{n}\right)\log\left[\frac{P\left({\hat{u}}_{m},{\hat{v}}_{n}\right)}{P\left({\hat{u}}_{m}\right)\cdot P\left({\hat{v}}_{n}\right)}\right], \end{array}$$

defining all terms as zero where *P*($\hat{u}$_*m*_) = 0 or $P\left({\hat{v}}_{n}\right)\;=\;0$.

Finally we compute the mean over all matrix elements (each ranging between 0 and 1 bit),

(8) $$\begin{array}{r} I\left({\text{u}}_{t},{\text{v}}_{t+1}\right)=\text{MEAN}{\left\{I_{mn}^{\left(uv\right)}\right\}}_{mn}=\frac{1}{MN}\overset{M}{\underset{m=1}{\sum }}\overset{N}{\underset{n=1}{\sum }}I_{mn}^{\left(uv\right)} \end{array}$$

This measure is applied in the present paper to quantify the mutual information *I*(**s**_*t*_, **s**_*t*+1_) between subsequent RNN states, as well as the mutual information *I*(**x**_*t*_, **s**_*t*+1_) between the momentary input and the subsequent RNN state.

## Data Availability Statement

The raw data supporting the conclusions of this article will be made available by the authors, without undue reservation.

## Author Contributions

All authors listed have made a substantial, direct, and intellectual contribution to the work and approved it for publication.

## Funding

This work was funded by the Deutsche Forschungsgemeinschaft (DFG, German Research Foundation): grant KR 5148/2-1 (project number 436456810) to PK.

## Conflict of Interest

The authors declare that the research was conducted in the absence of any commercial or financial relationships that could be construed as a potential conflict of interest.

## Publisher's Note

All claims expressed in this article are solely those of the authors and do not necessarily represent those of their affiliated organizations, or those of the publisher, the editors and the reviewers. Any product that may be evaluated in this article, or claim that may be made by its manufacturer, is not guaranteed or endorsed by the publisher.
